# Health-Related Quality of Life in Bariatric and Metabolic Surgery

**DOI:** 10.1007/s13679-020-00392-z

**Published:** 2020-06-18

**Authors:** Karen D. Coulman, Jane M. Blazeby

**Affiliations:** 1 Population Health Sciences, Bristol Medical School, University of Bristol, 1-5 Whiteladies Road, Bristol, BS8 1NU UK; 2 Obesity and Bariatric Surgery Service, North Bristol NHS Trust, Bristol, UK; 3 Division of Surgery, Head and Neck, University Hospitals Bristol NHS Foundation Trust, Bristol, UK; 4 National Institute for Health Research Bristol Biomedical Research Centre Surgical Innovation Theme, Bristol Centre for Surgical Research, Bristol Medical School: Population Health Sciences, University of Bristol, Bristol, UK

**Keywords:** Bariatric surgery, Obesity, Health-related quality of life, Psychosocial outcomes

## Abstract

**Purpose of Review:**

This review describes the latest evidence for the impact of bariatric surgery on health-related quality of life (HRQL).

**Recent Findings:**

The impact of bariatric surgery on HRQL is less well-understood than its clinical effectiveness on weight and co-morbidities. Poor-quality study design and different HRQL measures challenge systematic reviews and meta-analyses. Available limited evidence suggests that physical aspects of HRQL may improve more than mental health aspects of HRQL after bariatric surgery, reaching maximal benefits 1–2 years post-surgery. Comparative HRQL analyses between bariatric procedures cannot be made due to a lack of randomised data. Qualitative research highlights the tensions patients experience after bariatric surgery, which provides insights to observed changes in HRQL.

**Summary:**

Standardized HRQL measures are being developed and agreed to improve future evidence synthesis. Two multi-centre randomised trials of bariatric surgical procedures including detailed HRQL assessment are in progress. It is hoped that the combination of comparative high-quality HRQL data and information from qualitative studies will provide new insights into patient well-being and health after bariatric surgery.

## Introduction

Over 650 million or 13% of adults worldwide are living with obesity (body mass index (BMI) ≥ 30 kg/m^2^), representing a tripling of figures since 1975 [1]. Obesity is associated with an increased risk of type 2 diabetes, cardiovascular disease, certain cancers, depression, reduced quality of life and premature death [2–7]. Effective public health initiatives are critically important to prevent future obesity; however, experts agree these are not sufficient to achieve weight loss in those already living with obesity, particularly those with severe and complex obesity (BMI of ≥ 40 kg/m^2^, or 35–40 kg/m^2^ with another significant health problem that could be improved by weight loss), who are at the highest risk of morbidity and premature death [8–10]. In 2018, 3% of adults in England were reported to have a BMI ≥ 40 kg/m2, and data from the USA indicate that 40% of the total healthcare costs of overweight and obesity can be attributed to the 8% of the US population with a BMI ≥ 35 kg/m^2^ [8, 10, 11]. Thus, government-supported public health initiatives are urgently needed to prevent people becoming obese as well as effective clinical interventions for those who have already become severely obese, to reduce associated morbidity and healthcare costs [8].

In people with severe and complex obesity, bariatric surgery is the most clinically effective treatment, leading to greater weight loss and improvement in control of type 2 diabetes, compared with lifestyle interventions or drug therapy alone [9, 12, 13]. The sleeve gastrectomy (SG) and Roux-en-Y gastric bypass (RYGB) are the most common bariatric operations carried out worldwide with the adjustable gastric band (AGB) decreasing in recent years, and the one-anastomosis gastric bypass (OAGB) now gaining popularity [14]. Current data show that proportions of each of these procedures are 46.0%, 38.2%, 5.0% and 7.6%, respectively [14]. Each of these procedures works slightly differently; mechanisms include restriction in the amount of food able to be consumed, reduction in hunger, improvement in satiety, shift in food preferences and altered gut hormones, bile acids and vagal signalling [15]. Although the clinical benefits of bariatric surgery are well-established, the impact of bariatric surgery on psychosocial outcomes such as health-related quality of life (HRQL) is less clear. The purpose of this review is to highlight the importance of psychosocial outcomes when evaluating interventions for obesity such as bariatric surgery, and to describe the latest evidence for the impact of bariatric surgery on HRQL.

## The Psychosocial Impact of Severe and Complex Obesity

The physical and metabolic burdens associated with severe and complex obesity are well-known; however, its psychosocial impact is also of critical importance [16•, 17, 18]. These issues may be explored with several methodologies including qualitative methods and assessment of patient-reported outcomes. A systematic review of qualitative studies of peoples’ motivations for bariatric surgery identified physiological, emotional, cognitive and interpersonal/environmental reasons for wishing to undergo surgery [16•]. People with severe and complex obesity suffer from social stigma and discrimination related to their weight which is in turn associated with negative physical and psychological outcomes [17–22]. These individuals are more likely to suffer with depression, anxiety, disordered eating, body image dissatisfaction and impaired HRQL [20, 21, 23,24, 25••]. In recognition of this, the British Obesity and Metabolic Surgery Society has recently published guidelines for psychological support pre- and post-bariatric surgery [26]. Given these psychosocial issues associated with severe obesity and their impact, interventions for severe and complex obesity should evaluate both psychosocial and clinical outcomes [27•, 28]. Psychosocial outcomes are often measured via patient self-report, using patient-reported outcome measures.

Patient-reported outcomes (PROs) may be defined as “any report of the status of a patient’s health condition that comes directly from the patient, without interpretation of the patient’s response by a clinician or anyone else” [29]. It is important to include PROs when evaluating health interventions as some outcomes are known only to the patient, for example body image, where there are no observable or physical measures [29]. Additionally, improvements in clinical outcomes may not always correspond with improvements in how the patient functions or feels, so PROs can be used to provide a unique patient perspective on the effects of a treatment [29]. PRO measures may assess one single aspect (or domain) of health (e.g. pain or depression) or assess several domains of health such as HRQL [29]. HRQL is a commonly measured PRO defined as “a multidomain concept that represents the patient’s general perception of the impact of an illness and its treatment on physical, psychological, and social aspects of life.” [29, 30]*.* Kolotkin and Anderson undertook a systematic review of systematic reviews examining the impact of obesity on HRQL [25••]. They concluded that people with BMI ≥ 40 kg/m^2^ and those seeking bariatric surgery had the greatest impairment in HRQL, with physical aspects (domains) of HRQL more related to the degree of obesity than mental domains, as assessed using the Short-Form-36 (SF-36) HRQL measure.

## What Do We Know About the Impact of Bariatric Surgery on HRQL?

Systematic reviews assessing the impact of bariatric surgery on HRQL have been hampered by poor-quality evidence due to few randomised controlled trials (RCTs) examining HRQL after bariatric surgery, and limited well-designed prospective observational studies with long-term follow-up of HRQL [25••, 28, 31–39]. Another issue is the huge number of different HRQL measures used in bariatric surgery studies, with systematic reviews identifying up to 68 (across 86 included studies) different measures [25••, 28]. This heterogeneity of HRQL measures limits the ability to make comparisons and undertake meta-analyses, making it difficult to draw conclusions about the impact of bariatric surgery on HRQL [28, 32, 35]. However, a few themes can be identified from systematic reviews.

### Impact of Bariatric Surgery on Generic HRQL

The SF-36 questionnaire has been the most common measure of HRQL used in bariatric surgery studies [28, 31–38]. This is a generic measure of HRQL which includes 36 items across eight domains (physical functioning, role-physical, bodily pain, general health, vitality, social functioning, role-emotional and mental health) which are scored individually and contribute to a physical component score (PCS) and a mental component score (MCS) (Table 1) [40, 41]. The SF-36 is widely used across clinical specialties and has been reported to take 10 min or less to complete [42]. Generic HRQL measures such as the SF-36 allow for comparison across clinical areas and with population norms [40]. Two previous studies, undertaken in Norway and Bahrain, investigated the validity of the SF-36 in people with severe obesity [43, 44]. The two summary scales (PCS and MCS) were found to have adequate validity in this population; however, the validity of the eight individual domains was less certain, with authors suggesting that PCS and MCS should be the primary endpoints when using the SF-36 in this population [43]. A conclusion drawn by the majority of systematic reviews examining the SF-36 in bariatric surgery is that the PCS improved more consistently after surgery than the MCS [25••, 32–39]. Reviews also reported that peak improvements in scores occur 1–2 years post-surgery followed by a gradual decline levelling off by 5 years, with levels still higher than pre-operative baseline [31, 33, 35].

**Table 1:** Domains and scoring of two commonly used measures to assess health-related quality of life in bariatric surgery studies

Instrument	Domains	Scoring
SF-36 (36 items)	Physical functioning	Provides scores for each of the eight domains as well as a physical component score (PCS) and a mental component score (MCS)
	Role-physical	
	Bodily pain	
	General health	
	Vitality	
	Social functioning	
	Role-emotional	
	Mental health	
IWQOL-Lite (31 items)	Physical function	Provides scores for each of the five domains as well as a total score of weight-related quality of life.
	Self-esteem	
	Sexual life	
	Public distress	
	Work	

*SF-36* Short Form-36 questionnaire, *IWQOL-Lite* Impact of Weight on Quality of Life-Lite questionnaire

These findings are supported by two recent American prospective cohort studies [45, 46]. The Utah Obesity Study examined 12-year changes in HRQL in people undergoing RYGB compared with people with severe obesity who sought (but did not undergo) surgery and those who did not seek bariatric surgery [45]. SF-36 PCS scores peaked at 2 years post-surgery followed by a gradual decline, with 12-year scores still higher than baseline and both comparison groups. Small improvements in MCS were seen at 2 years post-surgery; however, these were not maintained at 6 or 12 years. Limitations of this study were the large amount of missing HRQL data at 12-year follow-up, with important baseline differences noted between completers and non-completers [45, 47]. The multi-centre Longitudinal Study of Bariatric Surgery (LABS) reported SF-36 scores up to 5 years post-surgery in 1529 people who underwent RYGB, SG and AGB (comparisons between procedures were not made) [46]. Clinically meaningful improvements in PCS were found at 1-year post-surgery followed by relatively stable levels between 1 and 5 years. Minimal changes from baseline were found in MCS; however, baseline MCS was similar to US norms, whereas baseline PCS was lower than US norms, which may have accounted for the differences. This aligns with findings that PCS is more strongly linked to obesity than MCS [25••]. Szmulewicz et al. assert that MCS only serves as a proxy for mental health conditions which are better captured using specific validated measures of mental health conditions; thus, MCS may not be sensitive to capture impairments in psychological and mental health [38].

### Impact of Bariatric Surgery on Obesity-Specific HRQL

A limitation of generic HRQL measures is they do not include aspects of HRQL specific to particular clinical areas, such as body image and social stigma in the case of people living with obesity, and thus may not be as sensitive to change in this population as an obesity-specific HRQL measure [48, 49]. Kolotkin and Andersen, who undertook an overview of systematic reviews in this area, concluded that post-surgical effect sizes were consistently larger with obesity-specific HRQL measures than the SF-36 [25••]. However, comprehensive comparisons are difficult to undertake given the range of different obesity-specific HRQL measures used across studies. The Impact of Weight on Quality of Life-Lite (IWQOL-Lite) is one of the more frequently used of these obesity-specific measures [25••]. This measure includes 31 items across five domains (physical function, self-esteem, sexual life, public distress, work) and provides a total score of weight-related quality of life (Table 1) [50]. The IWQOL-Lite was developed and validated for use in people with severe obesity [50, 51]. In the Utah Obesity Study, improved IWQOL-Lite scores mirrored those of the SF-36 PCS—with peak improvements seen at 2 years post-RYGB followed by a gradual decline that was still improved compared with baseline and control groups at 12 years [45]. Recently, the IWQOL-Lite Clinical trials (CT) version has been developed to comply with US Food and Drug Administration guidance for patient-reported outcomes [29, 52, 53]. This newer and shorter version (20 items) is based on extensive qualitative work with a wider variety of individuals living with obesity and was found to have good acceptability in this population [52].

### Comparisons of Improvements in HRQL Across Different Types of Bariatric Surgery

A few reviews have attempted to compare HRQL across different types of bariatric surgery procedures; however, none of these undertook a meta-analysis. All acknowledged heterogeneity of measures used and poor quality of studies as limitations [31, 32, 35, 37]. A recent large Dutch multi-centre cohort study examined RAND-36 scores (nearly identical to the SF-36, measuring the same domains, standardly used in Dutch bariatric hospitals) 1 year after RYGB and SG [54]. They found greater improvements in the physical functioning and general health perception domains for RYGB compared with SG; however, the authors acknowledged that these differences could be explained by important baseline differences (selection bias) between those that underwent SG versus RYGB. The lack of well-designed and conducted RCTs with long-term follow-up means that true comparative assessments of RYGB, SG and AGB are missing from the literature. A UK multi-centre RCT (the By-Band-Sleeve study) with a co-primary endpoint of weight loss and HRQL at 3 years has recently completed recruitment (*n* = 1351) [55••]. HRQL measures include both generic (the EuroQoL-5D (EQ-5D) and the SF-12—a shorter version of the SF-36) and specific measures such as the IWQOL-Lite [56] and others assessing gastrointestinal complications and anxiety and depression. This will be the first large-scale pragmatic study comparing all three procedures that includes a comprehensive assessment of HRQL. The Scandinavian BEST (Bypass Equipoise Sleeve Trial) study is an ongoing multi-centre registry-based RCT comparing RYGB and SG, with a target sample size of 2100 patients [57••]. The study has a co-primary endpoint of severe adverse events and percentage weight loss at 5 years, with a number of secondary endpoints including HRQL as measured by the EQ-5D, the SF-36 and the Obesity Problems (OP) scale, an obesity-specific HRQL measure validated in the Scandinavian population [58, 59].

## What Are the Methodological Issues to Be Addressed with HRQL Assessment in Bariatric Surgery?

Standardization of HRQL measures used in future bariatric surgery studies is needed to overcome the current issue of heterogeneity of measures leading to difficulties synthesizing HRQL results of individual studies. To improve outcome selection and reporting in future bariatric surgery effectiveness trials, the UK-based BARIACT study developed a core outcome set for bariatric surgery, using a Delphi process with health professionals and patients [60]. HRQL was one of the nine items prioritised for inclusion in the final core outcome set. Building upon the BARIACT study, work is underway to standardize measures of HRQL through the Standardizing Quality of life measures in Obesity Treatment (SQOT) initiative, an international collaboration of healthcare professionals and people living with obesity aiming to achieve global consensus on the key components of HRQL and preferred measures [61, 62]. This may include a recommendation to include both a generic *and* an obesity-specific HRQL measure. This would allow information about specific issues relevant to people with obesity to be compared with generic HRQL issues which could also be considered in relation to population norms [25••, 28]. More well-designed RCTs with long-term follow-up are needed to provide good quality evidence comparing the impact of different bariatric surgery operations on HRQL [25••]. The By-Band-Sleeve study (*n* = 1351) and the BEST study (*n* = 2100), both ongoing, will be the largest randomised datasets [55••, 57••].

Given the prioritisation of HRQL as a “core” outcome of bariatric surgery, HRQL should also be included as part of routinely collected bariatric surgery registry data to inform health policy [25••, 60]. HRQL should be measured at baseline (pre-surgery) and followed-up long-term post-surgery, as for clinical outcomes, so that clinical and HRQL data can be considered in relation to each other [28]. To reduce response bias, HRQL measures should be completed directly by patients themselves “without interpretation of the patient’s response by a clinician or anyone else” [29]. The numbers of patients providing HRQL data at each timepoint and reasons for missing data should be documented, as HRQL data is often not missing at random; for example, patients with poorer outcomes may not return HRQL measures [63–65]. In general, response rates of ≥ 80% are considered to be representative of the full sample, with < 60% sometimes being referred to as unrepresentative [66]. The International Federation for the Surgery of Obesity and Related Disorders (IFSO) has initiated a global registry project to standardize the outcomes used to evaluate bariatric surgery on an international level to allow for comparisons of outcomes across countries with the aim of improving patient outcomes [67]. Together with the SQOT initiative, these projects will develop consensus on the most appropriate HRQL measures to evaluate bariatric surgery, and the timepoints at which HRQL (and other outcomes) should be measured post-surgery. The next challenge will be how to communicate HRQL information obtained from clinical trials and registry data alongside clinical data to patients when making decisions about undergoing bariatric surgery.

## The Role of Qualitative Research in Assessing the Psychosocial Impact of Bariatric Surgery

The patient’s perspective of psychosocial outcomes of bariatric surgery can be investigated using qualitative research methods. Qualitative research seeks to understand how people view, experience and make sense of their social world [68–70]. In the context of health research, qualitative research seeks to ask the “what” and “why” questions, rather than “how big” or “how many” that quantitative research seeks to answer [68]. Data are usually collected in a face-to-face setting through observation of behaviour and/or interacting with informants to seek their views, for example through semi-structured interviews [68, 70]. Qualitative research with patients can provide complementary information about the patient’s experience of psychosocial outcomes of bariatric surgery [68, 71]. Questionnaire studies using HRQL measures can sample a larger number of participants than can be included in a qualitative study; however, qualitative research can explore patients’ perspective of outcomes in greater depth to help understand complexities [68]. This can help to explain findings from HRQL studies including any inconsistencies across studies.

A systematic review and synthesis of qualitative research studies investigating the patient perspective of living with the outcomes of bariatric surgery was previously carried out by our team [27•]. This synthesis highlighted themes of control, normality and ambivalence in living with bariatric surgery across different areas of health and life (weight, activities of daily living, physical health, psychological health, social relations, sexual life, body image, relationship with food). The impact on physical health and activities of daily living was generally positive; however, there were more tensions in the other aspects of health. For example, the review highlighted that patients in the included studies reported some psychological benefits including reduced depression and improved self-confidence, however, also experienced challenges in establishing a new identity and acquiring new coping strategies to replace food. The results of the synthesis help to provide more insight into findings from studies using the SF-36 that MCS does not improve as consistently as PCS after bariatric surgery. Qualitative research is increasingly being nested within large multi-centre trials, such as the By-Band-Sleeve study [56]. This embedded qualitative research is recognised to help with recruitment and other trial processes but can also help with the understanding of trial participants’ experiences and help to explain trial findings such as HRQL [72].

## Conclusions

Severe and complex obesity has a negative impact on the psychosocial aspects of health, including HRQL. These aspects of health are as important to consider as clinical outcomes when evaluating interventions to treat severe and complex obesity. Bariatric surgery is the most clinically effective treatment for severe and complex obesity related to weight loss and reduction of co-morbidities. The impact of bariatric surgery on HRQL is less clear-cut. HRQL is often measured using PRO measures. These include generic HRQL measures, such as the SF-36, which are widely used and allow for comparisons with community norms. The SF-36 is the most commonly used HRQL measure in bariatric surgery studies. Studies suggest that the physical components of HRQL may improve more than the mental components, and that peak improvements in HRQL occur 1–2 years after surgery followed by a levelling off by 5 years. It is important to note that data at 5 years are still better than baseline estimates. Obesity-specific measures, however, such as the IWQOL-Lite, may be more sensitive to change in people with severe and complex obesity. These appear to show greater effect sizes than generic HRQL measures after bariatric surgery. However, comparisons of studies have been difficult to undertake due to poor-quality study design and heterogeneity of HRQL measures. These problems limit the ability to make comparisons across different types of bariatric operations. Two large multi-centre RCTs with comprehensive HRQL assessments comparing different types of bariatric procedures are currently in progress which will provide high-quality evidence. The international SQOT initiative building on the BARIACT project is working to standardize HRQL measures to be used in studies evaluating treatments for obesity, which will improve the comparability of future evidence. Psychosocial outcomes of bariatric surgery have also been investigated using qualitative research methods which have helped to provide more depth on the complexities of HRQL change after bariatric surgery. High-quality randomised HRQL data with embedded qualitative research will help to build the evidence base and understanding in this area.
